# Single-cell transcriptomic landscape reveals the role of intermediate monocytes in aneurysmal subarachnoid hemorrhage

**DOI:** 10.3389/fcell.2024.1401573

**Published:** 2024-09-10

**Authors:** Ningqin Meng, Ying Su, Ziming Ye, Xufeng Xie, Ying Liu, Chao Qin

**Affiliations:** ^1^ First Affiliated Hospital, Guangxi Medical University, Nanning, China; ^2^ The first people’s hospital of Yulin, Guangxi, China; ^3^ Department of Rehabilitation, The First Affiliated Hospital of Guangxi Medical University, Nanning, Guangxi, China

**Keywords:** ScRNA-seq, aneurysmal subarachnoid hemorrhage, monocytes, neuroinflammation, TLR

## Abstract

**Objective:**

Neuroinflammation is associated with brain injury and poor outcomes after aneurysmal subarachnoid hemorrhage (SAH). In this study, we performed single-cell RNA sequencing (scRNA-seq) to analyze monocytes and explore the mechanisms of neuroinflammation after SAH.

**Methods:**

We recruited two male patients with SAH and collected paired cerebrospinal fluid (CSF) and peripheral blood (PB) samples from each patient. Mononuclear cells from the CSF and PB samples were sequenced using 10x Genomics scRNA-seq. Additionally, scRNA-seq data for CSF from eight healthy individuals were obtained from the Gene Expression Omnibus database, serving as healthy controls (HC). We employed various R packages to comprehensively study the heterogeneity of transcriptome and phenotype of monocytes, including monocyte subset identification, function pathways, development and differentiation, and communication interaction.

**Results:**

(1) A total of 17,242 cells were obtained in this study, including 7,224 cells from CSF and 10,018 cells from PB, mainly identified as monocytes, T cells, B cells, and NK cells. (2) Monocytes were divided into three subsets based on the expression of CD14 and CD16: classical monocytes (CM), intermediate monocytes (IM), and nonclassical monocytes (NCM). Differentially expressed gene modules regulated the differentiation and biological function in monocyte subsets. (3) Compared with healthy controls, both the toll-like receptor (TLR) and nod-like receptor (NLR) pathways were significantly activated and upregulated in IM from CSF after SAH. The biological processes related to neuroinflammation, such as leukocyte migration and immune response regulation, were also enriched in IM. These findings revealed that IM may play a key role in neuroinflammation by mediating the TLR and NLR pathways after SAH.

**Interpretation:**

In conclusion, we establish a single-cell transcriptomic landscape of immune cells and uncover the heterogeneity of monocyte subsets in SAH. These findings offer new insights into the underlying mechanisms of neuroinflammation and therapeutic targets for SAH.

## Introduction

Aneurysmal subarachnoid hemorrhage (SAH) is a devastating stroke resulting from the rupture of intracranial aneurysm (Suarez et al., 2006). Although SAH only accounts for about 5–10 percent of all strokes, it is characterized by high disability and mortality (Rinkel and Algra, 2011). About 20 percent of patients who receive timely treatment and survive will develop significant neurological dysfunction after SAH, leading to a dramatic decline in quality of life (Nieuwkamp et al., 2009). It is important to note that the age of patients with SAH is much younger than those with ischemic stroke, which also imposes a significant health burden on society (Lawton and Vates, 2017; Robba et al., 2024). Unfortunately, the mechanisms responsible for the high mortality and morbidity are not yet completely understood. Accumulating evidence suggests that neurological complications, especially early brain injury, cerebral vasospasm, and delayed cerebral ischemia (DCI) (Wu et al., 2022; Dodd et al., 2021), are strongly associated with unfavorable outcomes in SAH (Thilak et al., 2024). And neuroinflammation has recently been identified as a crucial contributor to the development of neurological complications (Zeyu et al., 2021; Lucke-Wold et al., 2016). Hence, a better understanding of neuroinflammation mechanisms allows health professionals to develop strategies to reduce the risk of neurological complications.

Neuroinflammation plays a crucial role in brain injury related to neurological complications, leading to a poor outcome after SAH. To date, the mechanisms of neuroinflammation after SAH remain unknown. Undoubtedly, the metabolites of red blood cells (RBC) play a key role in the development of neuroinflammation (Bulters et al., 2018; Pan et al., 2020). After SAH onset, the hematoma, mainly comprised of RBC and its metabolites, deposits in the subarachnoid space and the brain’s surface. The metabolites of RBC, such as hemoglobin, free heme, and iron ions, act as danger-associated molecular patterns (DAMPs) to activate immune cells and signaling pathways, causing inflammatory cascade reaction (Khey et al., 2020; Kwon et al., 2015; Coulibaly and Provencio, 2020). Previous studies have described in detail how the resident (microglia and astrocytes) and peripheral immune cells (monocytes and neutrophils) are involved in brain injury in SAH (Coulibaly and Provencio, 2020). Still, these studies cannot fully elucidate the mechanisms of neuroinflammation. The priority is to find which kinds of immune cells are the critical contributors to neuroinflammation. It is encouraging to see that more and more studies have found monocytes may play an indispensable role in the development of neuroinflammation after SAH.

Monocytes, produced primarily in the bone marrow, circulate in the circulatory system as peripheral immune cells and eventually migrate into tissue for innate immunity: phagocytosis, antigen presentation, and cytokine production (Narasimhan et al., 2019). Monocytes continue to increase for 6–8 days, and monocytosis is associated with delayed cerebral ischemia and poor functional outcomes after SAH (Gusdon et al., 2021). In addition, compared with PB, the proportion of intermediate monocytes is higher in CSF in SAH (Moraes et al., 2015). Monocytes migrate into the brain and participate in neuroinflammation, leading to intracranial cerebral vasospasm (Jackson et al., 2021). The monocytes from patients with SAH express higher mRNA levels of CXCL10 than those from patients with non-SAH, which shows its potential significance as a therapeutic target (Sanchez et al., 2024). Nonclassical monocytes mediate neuroinflammation driven by various chemokines in the brain, and the monocyte activation profile is a potential target for immunosuppressive therapy after SAH (Mohme et al., 2020). These studies have demonstrated that monocytes play a significant role in the development of neuroinflammation following SAH.

However, these studies mentioned above on monocytes are mainly limited to the dynamic changes of cytology or inflammatory factor profile, and little is known about the heterogeneity of transcriptome and phenotype across monocyte subsets. Thus, exploring the transcriptome characteristics and biological functions of monocytes after SAH helps understand the molecular mechanisms of neuroinflammation. In this study, we used scRNA-seq to study the underlying mechanisms of neuroinflammation following SAH at the genetic level.

## Methods

### Human subjects

Two male patients with SAH were enrolled in this study. Paired CSF and PB samples were collected from each patient on day 7 after onset through lumbar drainage. The inclusion criteria for patients with SAH are as follows: (1) age ≥18 years old; (2) sudden headache accompanied by epilepsy and other clinical manifestations; (3) aneurysm subarachnoid hemorrhage was diagnosed through computed tomography examination and digital subtraction angiography on admission. The exclusion criteria were listed as follows: (1) patients with systemic inflammatory diseases such as intracranial infection, urinary tract infection, and other infectious diseases; (2) subarachnoid hemorrhage caused by other diseases, such as arteriovenous malformation, hemorrhagic stroke, and brain trauma; (3) patients using immunosuppressive drugs. This study was approved by the ethics committee of the First Affiliated Hospital of Guangxi Medical University and was conducted in accordance with the Declaration of Helsinki. Informed consent was obtained from the legally authorized representative. In addition, scRNA-seq data for CSF from eight healthy individuals were downloaded from the Gene Expression Omnibus database (GSE134578), which served as healthy control (HC) (Gate et al., 2020). The baseline demographic and clinical data for the healthy controls and patients with SAH were shown in Supplementary Tables S1 and S2.

### Isolation of mononuclear cells (MC)

CSF and PB samples were processed within half an hour after collection. MC was isolated from CSF and PB samples by using Ficoll-Paque PLUS (Solarbio Biotech, China). Briefly, samples diluted with phosphate-buffered saline (PBS) (Solarbio Biotech, China) were layered onto the Ficoll-Paque PLUS, followed by centrifugation and isolation of the buffy coat. The MC suspensions isolated from the buffy coat and mixed with cryoprotectant were frozen for 24 h in an ultralow temperature freezer and then transferred to liquid nitrogen for storage. The MC suspensions were thawed and quality-controlled prior to scRNA-seq.

### Single-cell RNA sequencing (10x genomics)

ScRNA-seq was performed on MC suspensions through the 10x Genomics Chromium platform according to the manufacturer’s instructions. In this study, eligible MC suspensions were loaded on a 10× Chromium microfluidics system to generate single-cell nanoliter-scale gel beads in emulsions (GEMs). Next, mRNA released from MC was reverse-transcribed into cDNA in every GEM. Finally, the cDNA from different GEMs were pooled and amplified by PCR to generate a cDNA library, which was sequenced using an Illumina NovaSeq 6,000.

### ScRNA-seq data processing and cell clustering

Cell ranger (10X Genomics) was performed to process raw data into gene expression matrices. Subsequently, Seurat was used for data filtering, normalization, dimensionality reduction, cell clustering (Butler et al., 2018). Low-quality cells were removed using the following criteria: (1) gene numbers <200 or >4,500; (2) total UMI counts <1,000 or >20,000; and (3) percentage of mitochondrial genes >10. Differentially expressed genes (DEGs) across clusters were explored using the Seurat function “FindAllMarkers” with parameters: min. pct = 0.25, logfc. threshold = 0.25. T-SNE (T-distributed Neighbor Embedding) was used to classify and visualize cell subsets (Kobak and Berens, 2019).

### Cell type identification

SingleR is an R package for automated cell type annotation in scRNA-seq data (Aran et al., 2019). In this study, SingleR and marker genes were used together to identify cell type identification. First, SingleR was performed to display a preliminary cell type annotation, which was subject to further manual identification based on the expression of marker genes and the following reference datasets: CellMarkrer, PanglaoDB, and Human Cell Atlas. Briefly, it is well known that the marker genes CD14 and CD16 are used for monocyte identification (Ziegler-Heitbrock et al., 2010; Ma et al., 2022; Villani et al., 2017; Wu et al., 2023; Shi et al., 2021). In scRNA-seq studies, CD3D and CD79A/CD79B have been recognized as reliable gene markers widely used for T (Menon et al., 2023; Wang et al., 2021; Zernecke et al., 2023; Zhu et al., 2020) and B cells (Tkachenko et al., 2023; Xu et al., 2023a) identification, respectively. In addition, NKG7 and GZMA are the markers for natural killer cells (NK) identification (Yao et al., 2023; Chen X. et al., 2023) and pro-platelet basic protein (PPBP) for platelet identification (Lee et al., 2023).

### Pseudotime trajectory analysis

Pseudotime trajectory analysis was performed to explore cell differentiation and development through the R software Monocle (Qiu et al., 2017). Monocytes with different colors were ordered on the pseudotime trajectory based on the differentially expressed genes. Monocle was used to construct monocyte lineage differentiation trajectory and identify differentially expressed gene modules that regulated the differentiation process.

### Enrichment analysis of function and signaling pathways

The Gene Ontology (GO) and Kyoto Encyclopedia of Genes and Genomes (KEGG) enrichment analysis were performed to study the heterogeneity of signaling pathways and biological functions across monocyte subsets. The GO annotation includes three categories: cellular component (CC), molecular function (MF), and biological process (BP). KEGG is a database resource for exploring high-level biological functions and signaling pathways. Gene set variation analysis (GSVA) was performed to study the activation of signaling pathways among monocyte subsets in an unsupervised way, showing the enrichment degree of target pathways across groups.

### Transcription factors and cell-cell communication interaction

Transcription factors are a class of proteins that regulate the transcription of the target gene. DoRothEA was used to evaluate the activation of transcriptional factors in different cell subsets (Garcia-Alonso et al., 2019). Cell-cell communication interaction can coordinate cell differentiation, immune regulation, and other life activities. Cellchat was used to explore cell-cell communication based on the expression of ligands, receptors, and their cofactors (Jin et al., 2021). The communication interaction networks between monocyte subsets and other immune cell types were observed through Cellchat.

## Results

### Single-cell transcriptomic landscape of immune cells after SAH

**FIGURE 1:**
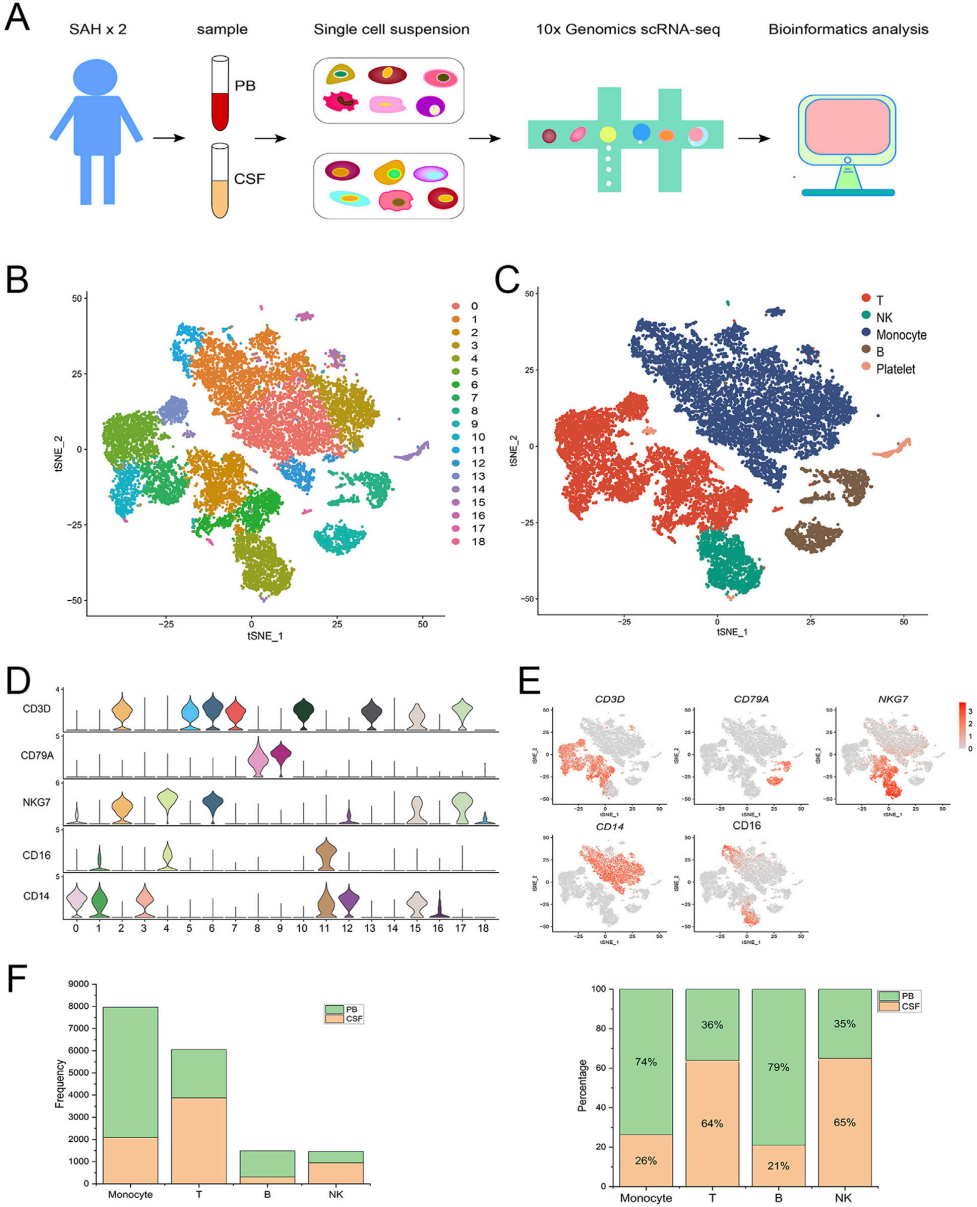
Single-cell transcriptomic landscape of immune cells after aneurysmal subarachnoid hemorrhage (SAH). **(A)** Overview of the experiment design and data analysis process. ScRNA-seq (10x Genomics) was performed on immune cells from CSF and PB samples corrected from SAH patients. **(B)** T-SNE visualization of 17,242 cells from CSF and PB. These cells were divided into 19 clusters (0–18). **(C)** The 19 cell clusters were further identified as 5 cell types: monocytes, T cells, B cells, natural killer cells, and platelets. **(D)** Violin plot showing the expression of the marker genes in each immune cell type. **(E)** The feature plot displaying the relative distribution of marker genes in each immune cell type, with low expression in gray and high expression in red. **(F)** The total number and percentage of each immune cell type from PB and CSF.

In this study, 4 samples were obtained from two male patients with SAH, including 2 CSF and 2 PB samples. Each sample was sequenced individually using 10x Genomics scRNA-seq (Figure 1A), and the gene expression matrices were pooled for downstream biological analysis. Finally, after the quality control, a total of 17,242 cells were obtained, including 7,224 cells from CSF and 10,018 cells from PB (Supplementary Figure S1). These 17,242 cells were divided into 19 (0–18) clusters by using T-SNE (Figure 1B). And then 19 cell clusters were further identified as four immune cell types and platelet, including monocytes (clusters 0, 1, 3, 11, 12, 15 and 16) with marker genes CD14 and CD16 (FCGR3A) (Ziegler-Heitbrock et al., 2010; Ma et al., 2022; Villani et al., 2017; Wu et al., 2023; Shi et al., 2021), T cells (clusters 2, 5, 6, 7, 10, 13 and 17) with marker gene CD3D (Menon et al., 2023; Wang et al., 2021; Zernecke et al., 2023; Zhu et al., 2020), B cells (clusters 8 and 9) with marker gene CD79A (Tkachenko et al., 2023; Xu et al., 2023a) and natural killer (NK) cells (clusters 4 and 18) with marker gene NKG7 (Figure 1C) (Yao et al., 2023; Chen X. et al., 2023). In addition, cluster 14 was defined as platelet (Lee et al., 2023), which was excluded from the subsequent analysis. The violin plot showed that each marker gene was specifically highly expressed in corresponding cell clusters (Figure 1D). The Feature plot displayed the relative distribution of marker genes across all clusters (Figure 1E). The histogram showed the total number and percentage of each immune cell type from PB and CSF (Figure 1F). Briefly, the 17,242 cells were identified as monocytes, T cells, B cells, NK cells, and platelets. Only monocytes were extracted for downstream analysis to explore the heterogeneity of transcriptome and phenotype across monocyte subsets after SAH.

### Identification of monocyte subsets

**FIGURE 2:**
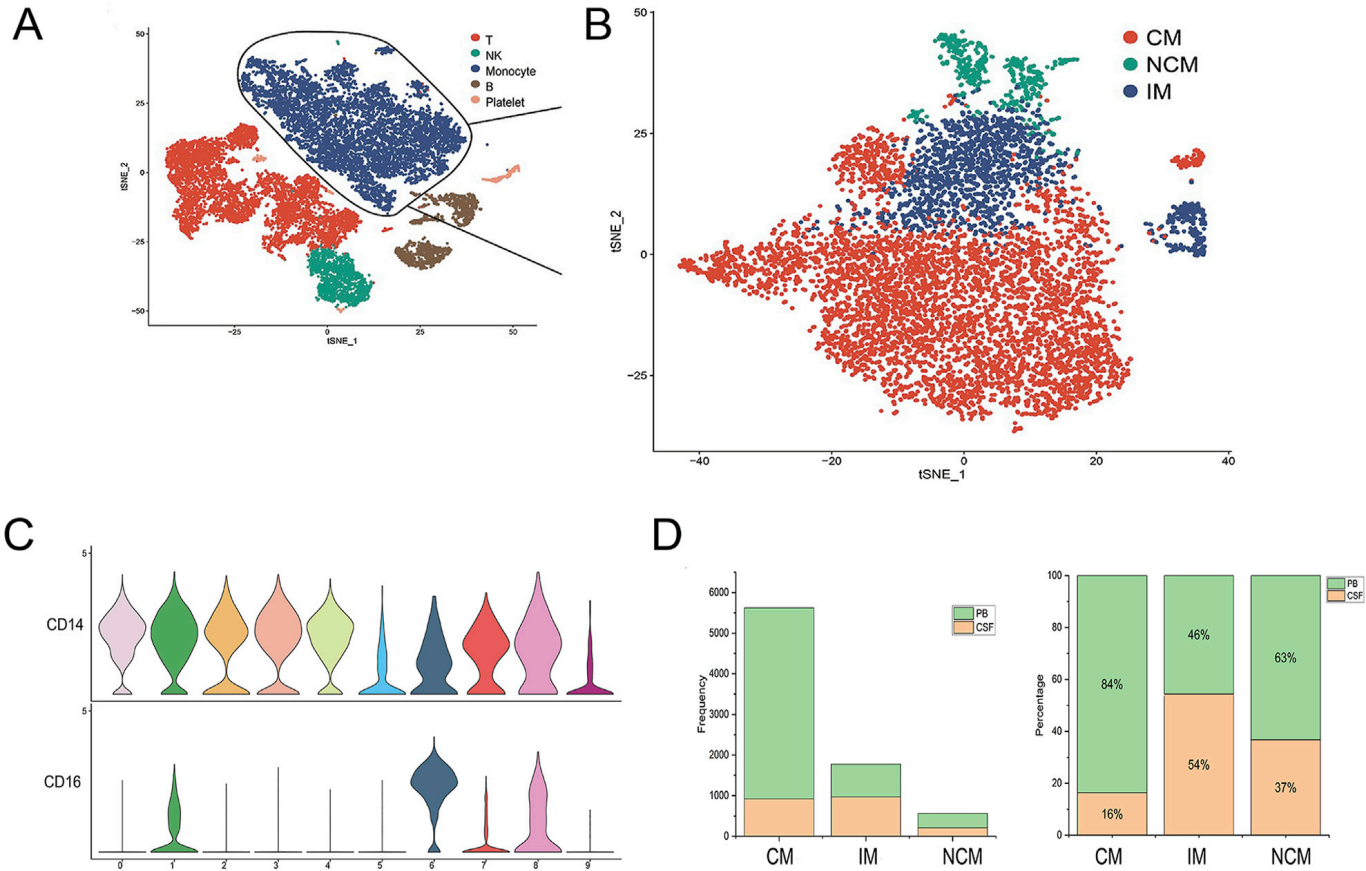
Identification of monocyte subsets. **(A, B)** Monocytes were divided into three subsets, including classical monocytes (CM), intermediate monocytes (IM), and nonclassical monocytes (NCM). **(C)** Violin plot showing the expression of marker genes across monocyte subsets. **(D)** The total number and percentage of monocyte subsets from PB and CSF.

Monocytes have multiple subtypes that differ in biological function, morphology, and transcriptional profile (Williams et al., 2023). To study monocyte heterogeneity, we further re-clustered monocytes. In this study, 2091 and 5,868 monocytes were obtained from CSF and PB samples, respectively (Figure 2A). Based on the expression levels of CD14 and CD16, these monocytes were traditionally divided into three subsets: classical monocytes (CM), intermediate monocytes (IM) and nonclassical monocytes (NCM) (Figure 2B). CM (CD14^++^ CD16^−^) highly expressed CD14 but no CD16, IM (CD4^++^CD16^+^) represented high expression of CD14 and low expression of CD16 while NCM (CD14^+^CD16^++^) showed high expression of CD16 together with low CD14 (Ziegler-Heitbrock et al., 2010). The violin plot showed the expression of marker genes CD14 and CD16 in each cell cluster (Figure 2C). The histogram showed the total number and percentage of monocyte subsets from PB and CSF (Figure 2D).

### Lineage differentiation trajectory of monocyte subsets

**FIGURE 3:**
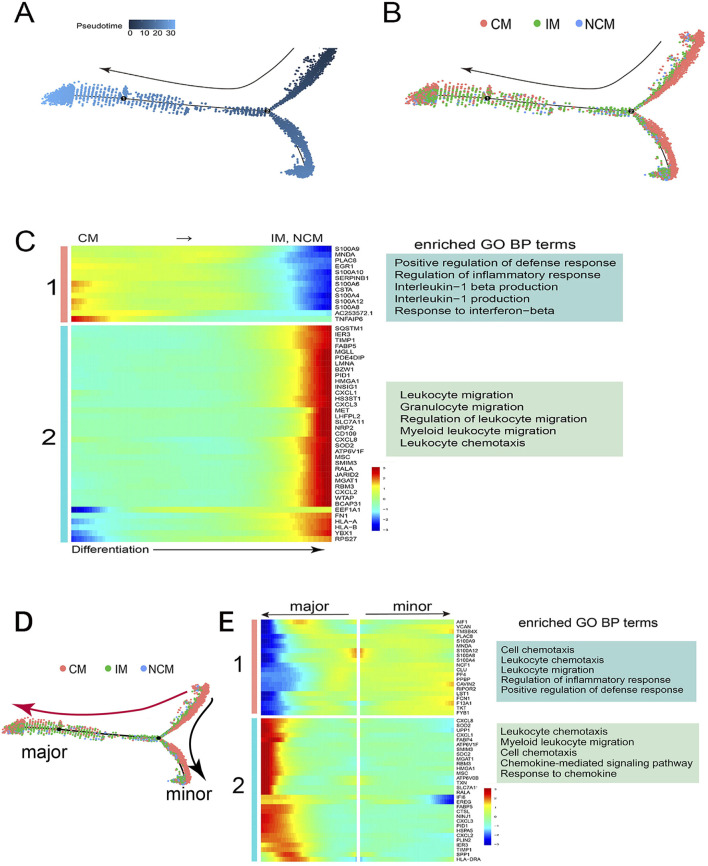
Gene regulation mechanism of monocyte differentiation. **(A)** Pseudotime trajectory of monocyte differentiation. Monocytes gradually differentiated from right to left, with a minor branch at the bottom right. **(B)** Monocyte subsets with different colors were mapped onto the trajectory, representing a lineage differentiation from CM to IM and NCM. **(C)** The heatmap showed that two gene modules, one and two, were significantly differentially expressed along the monocyte differentiation, and their representative GO biological processes were listed on the right. **(D)** The differentiation trajectory had two branches: the major (red Arrow) and the minor (black Arrow). **(E)** Gene module one exhibited high expression along the minor branch, whereas Gene module two displayed high expression along the major branch. Their respective representative GO biological processes were shown on the right.

Monocle was performed to map the differentiation trajectory of monocyte subsets and study the differentially expressed gene modules to explore the mechanisms of monocyte differentiation. Monocyte differentiation began on the right of the trajectory and progressed gradually to the left, with a minor branch at the bottom right. (Figure 3A). Monocyte subsets with different colors were mapped onto the trajectory, representing a gradual lineage differentiation transition from CM to IM and NCM (Figure 3B).

The heatmap showed that two key gene modules were significantly differentially expressed along the differentiation process of monocyte subsets (Figure 3C). Gene module one, containing the S100 gene family, showed high expression in CM but low expression in IM and NCM. GO enrichment analysis found that gene module one was related to the regulation of inflammatory response, the production of interleukin, and other biological processes (Figure 3C). Conversely, gene module two, including the CXCL gene family, was gradually highly expressed in IM and NCM along with differentiation, associated with immune cell migration and leukocyte chemotaxis (Figure 3C).

The differentiation trajectory had two branches, including the major branch (red Arrow) and the minor branch (black Arrow) (Figure 3D). Most of IM and NCM were mapped onto the major branch, while CM was mapped onto the minor branch. The heatmap revealed two differentially expressed gene modules along the differentiation trajectory (Figure 3E). Gene module one was highly expressed in monocytes differentiated towards the minor branch, which was related to regulation of inflammatory response and positive regulation of defense response (Figure 3E). However, Gene module two was significantly upregulated in monocytes differentiated towards the major branch, which was associated with immune cell chemotaxis and response to chemokine, etc (Figure 3E).

These monocyte subsets showed different gene expression patterns along the differentiation process, displaying significant transcriptome profile and phenotype heterogeneity. Together, these results suggested the differentially expressed gene modules may play a key role in the differentiation and biological functions of monocyte subsets.

### Monocytes mediated neuroinflammation after SAH

**FIGURE 4:**
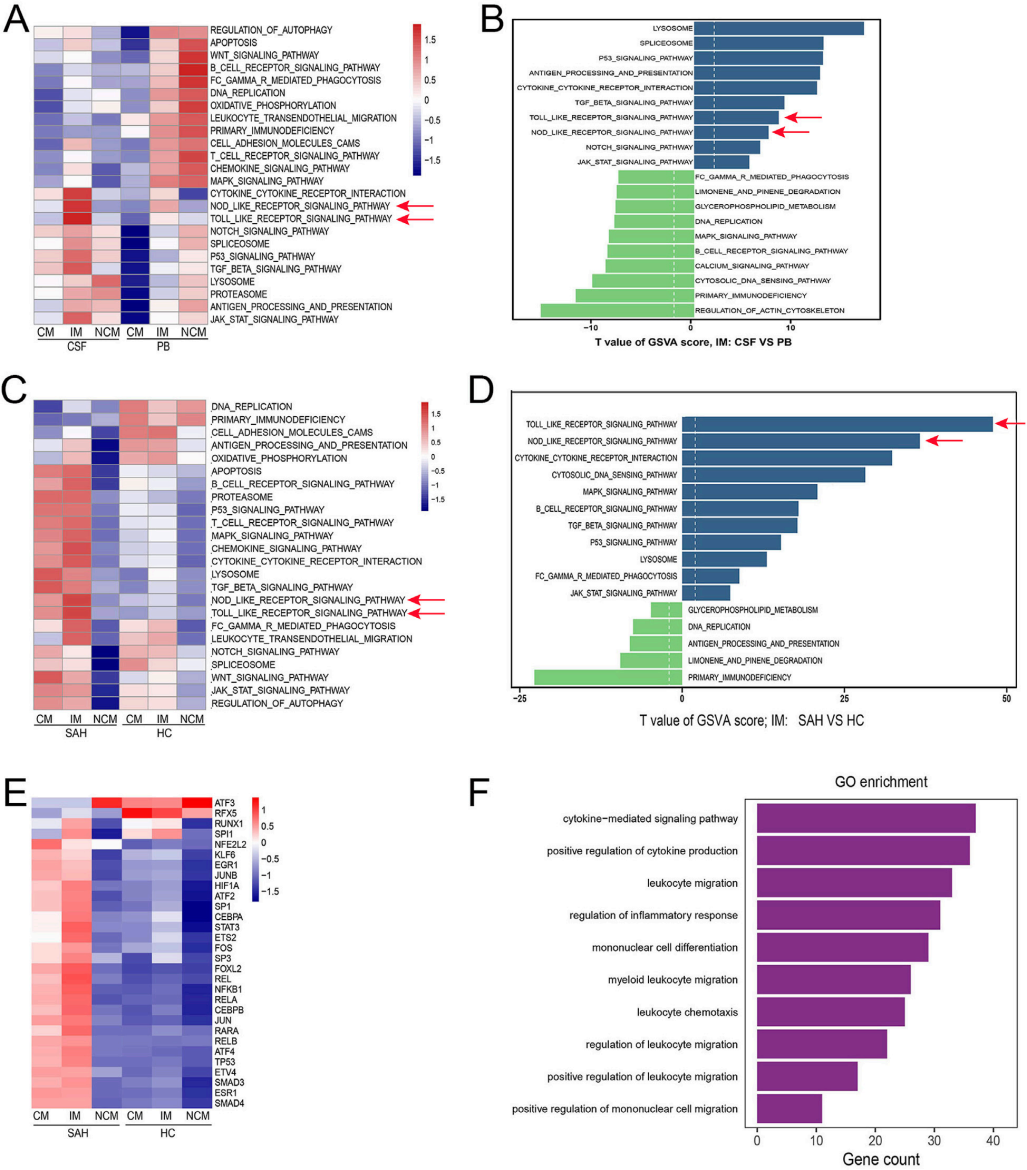
IM may play a key role in neuroinflammation after SAH. **(A)** Compared with PB, many signaling pathways were highly activated in IM from CSF, especially the TLR and NLR (red Arrow). The one above the line represents the monocyte subsets (CM, IM, and NCM), and the one below represents sample types (CSF and PB). **(B)** Compared to PB, the TLR and NLR pathways showed significant upregulation in IM from CSF (red arrow). The blue bars represent upregulated pathways, and the green bars represent downregulated pathways. **(C, D)** TLR and NLR pathways were significantly activated and upregulated in IM from CSF after SAH compared with HC (red Arrow). **(E)** Compared with HC, the activation of transcription factors was also considerably upregulated in IM from SAH. **(F)** GO biological processes related to neuroinflammation were enriched in IM from CSF after SAH.

GSVA was performed to study how the monocytes mediated neuroinflammation. First, the findings indicated that Toll-like receptor (TLR), Nod-like receptor (NLR), and other inflammation-related signaling pathways were more highly activated in IM from CSF compared with PB (Figure 4A, Arrow). Additionally, further comparative analysis demonstrated that these signaling pathways are significantly upregulated in IM from CSF (Figure 4B, Arrow). To explore whether these pathways were involved in neuroinflammation after SAH, we downloaded scRNA-seq data regarding CSF from eight healthy individuals to serve as healthy controls (HC). The frequency and percentage of monocyte subsets from CSF in both HC and SAH groups were shown in Supplementary Figure S2. Similarly, compared with HC, these pathways were significantly activated (Figure 4C, Arrow) and upregulated (Figure 4D, Arrow) in IM from CSF after SAH. In particular, the TLR and NLR pathways exhibited the highest activation and upregulation among these signaling pathways, which have been proven to be involved in neuroinflammation after SAH (Peng et al., 2024; Peng et al., 2023; Lai et al., 2023; Wang W. et al., 2023). In addition, the activation of transcription factors in IM increased after SAH (Figure 4E). To explore the changes in gene expression in IM, we conducted a comparative analysis of differentially expressed genes (DEGs) in HC and SAH. We found that many inflammation-related genes, especially those from the CCL and CXCL gene families, were significantly upregulated in SAH compared to HC (**Supplement DEGs**). GO enrichment analysis based on the DEGs showed that various critical biological processes related to neuroinflammation were enriched in IM after SAH (Figure 4F). To conclude, these findings suggest that IM may play a key role in regulating neuroinflammation by mediating the TLR and NLR pathways after SAH.

### Communication interaction was enhanced among monocyte subsets

**FIGURE 5:**
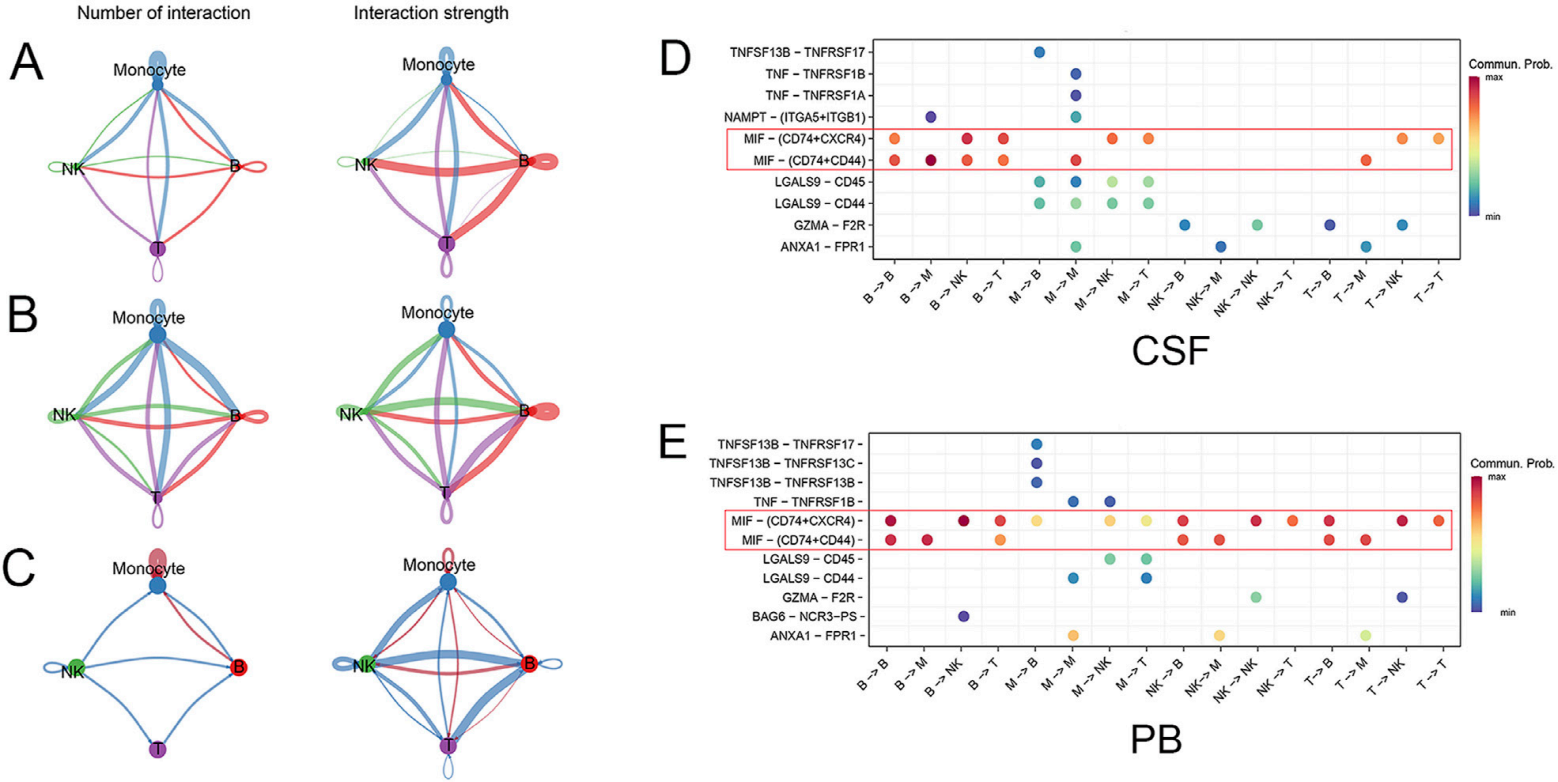
The communication interaction between different cell types. **(A)** The number and strength of communication interactions among various immune cell types in CSF. The line color matches the cell type, and the thickness indicates the intensity of communication interaction. **(B)** The number and strength of communication interactions among immune cell types in PB. **(C)** Compared with PB, the number and strength of communication interaction among monocyte subsets were significantly increased in CSF. The red line indicates upregulation, while the blue line indicates downregulation. **(D, E)** The communication probability of ligand-receptor pairs was different between CSF and PB. The MIF- (CD74+CXCR4) and MIF- (CD74+CD44) pairs were highly activated in both CSF and PB (red box).

Immune cells maintain immune system homeostasis through cell communication interaction. Cellchat was performed to study the cell communication network between various immune cell types. There was abundant cell communication interaction between the immune cells in CSF, especially monocyte subsets (Figure 5A). Likewise, communication interaction among various immune cell types was also complex and abundant in PB (Figure 5B). Compared with PB, the number and strength of communication interaction between monocyte subsets were significantly increased in CSF (Figure 5C). Notably, the communication interaction patterns of ligand-receptor pairs among these immune cell types in CSF and PB were different (Figure 5D, E). The macrophage migration inhibitory factor (MIF)- (CD74+CXCR4) and MIF- (CD74+CD44) pairs were highly activated among various immune cell types in both CSF and PB (Figure 5D, E, red box). In short, the communication interactions of monocyte subsets exhibited significant differences in CSF and PB, possibly due to varying immune microenvironments in SAH.

## Discussion

This study depicted the single-cell transcriptomic landscape of immune cells and explored the heterogeneity of transcriptome and phenotype across monocyte subsets in SAH through scRNA-Seq. Monocytes were divided into three subsets: CM, IM, and NCM, with a significant difference in their transcriptome profile, activation status, and biological functions. The differentially expressed gene modules could be the key factors in regulating the differentiation of monocyte subsets. The differentiation of monocyte subsets was a relatively stable development process, with a lineage starting from CM to IM and then NCM. Compared with HC, the TLR and NLR pathways were significantly activated and upregulated in IM from CSF after SAH. The biological processes related to neuroinflammation, such as leukocyte migration and immune response regulation, were also enriched in IM from CSF. This study highlighted that IM may play a key role in neuroinflammation by mediating the TLR and NLR pathways after SAH.

The breakdown products of RBC in subarachnoid space cause robust neuroinflammation immediately after SAH. Until now, the mechanisms of neuroinflammation after SAH are still unclear, which is the crucial reason why clinical interventions fail to have a good clinical outcome. Neuroinflammation following SAH is characterized by an imbalance in the immune microenvironments, such as the overactivation of immune cells, dysfunction of signaling pathways, and overproduction of inflammatory factors. The resident and peripheral immune cells, such as microglia, astrocytes, monocytes, and neutrophils, are highly activated and mediate neuroinflammation after SAH (Coulibaly and Provencio, 2020; Romoli et al., 2023). A wide range of inflammatory pathways are overactivated during the inflammatory response following SAH, particularly the TLR and NLR pathways (Lauzier et al., 2023; Jin J. et al., 2022; Hu et al., 2021). Dysregulated pathways lead to overactivation of immune cells, causing excessive production of inflammatory cytokines, interleukins, and tumor necrosis factors (He et al., 2024; Devlin et al., 2022; Chai et al., 2023). The crosstalk between monocyte overactivation and dysfunction of inflammatory pathways is a key contributor to the complexity of SAH pathophysiology. Among these activated pathways, the TLR and NLR are the most investigated pathways and have been proven to be involved in the development of neuroinflammation (Wang et al., 2022; Jin L. et al., 2022; Xu et al., 2021). However, few studies have been conducted on monocytes regarding the role of TLR and NLR pathways in SAH. In our study, both the TLR and NLR pathways were significantly activated and upregulated in IM from CSF compared to HC. Although many signaling pathways were also activated, the TLR and NLR pathways showed the highest level of activation and enrichment, indicating their key roles in inflammatory response after SAH. Similarly, a scRNA-seq study also indicates that monocytes play a significant role in brain injury following SAH (Wang X. et al., 2023). However, what is different from ours is that their study shows that the STAT3/Bcl-2 pathway, but not the TLR and NLR pathways, is the main cause of meningeal lymphatic vessel dysfunction. The discrepancy in conclusions between the two studies may be attributed to differences in samples and research methodologies. Consistent with our findings, another study demonstrates that the NLR pathway in monocytes is significantly upregulated, potentially leading to systemic inflammation and a poor clinical outcome (Díaz-García et al., 2023). Additionally, monocytes expressing high levels of CXCL10 play crucial roles in the inflammatory response, indicating it is a potential treatment target for future studies (Sanchez et al., 2024). Interestingly, our study also found that monocytes expressed a high level of CXCL gene family (Supplement DEGs), supporting the potential role of monocytes in leukocyte recruitment and migration in SAH. Monocytes may infiltrate the brain to help the recovery of neurological function (Chen H. et al., 2023), which is contrary to most previous studies. These studies highlight the significant roles of monocytes in neuroinflammation, although the mechanism by which they contribute remains unclear. In our study, we further demonstrated many transcription factors, such as STAT3 and NF-kB, were highly activated in IM from CSF, which had been proven to be involved in neuroinflammation (Liu et al., 2023; Samraj et al., 2014). Besides these, IM from CSF also played an essential role in leukocyte migration and immune response regulation after SAH. Collectively, our findings highlighted that IM may play a key role in neuroinflammation by mediating the TLR and NLR pathways, which could be a therapeutic target for SAH. Although our study is the first time to describe how IM regulates neuroinflammation at the gene level, much is still unknown about their biological functions in SAH. For now, further exploration is needed to determine whether IM eventually performs the roles of anti-inflammation or pro-inflammation, or whether they have some unknown biological characteristics in SAH.

Monocyte subsets show remarkable heterogeneity in transcriptomic profile and biological functions. CM is mainly responsible for phagocytosis and migration, IM is associated with antigen processing and presentation, while NCM is related to the surveillance of vasculature and anti-viral response (Gren et al., 2015; Kapellos et al., 2019). Our study found that gene module one was highly expressed in CM while gene module two was gradually expressed in IM/NCM along with differentiation trajectory. CM mainly showed high expression of the S100 family (S100A4, S100A8, S100A9, and S100A12), which was associated with the inflammatory response, production of interleukin, and interferon response. IM/NCM expressed high levels of the CXCL family (CXCL1, CXCL2, CXCL3, and CXCL8) and HLA-DRA was responsible for leukocyte migration and chemotaxis. Although these monocyte subsets display different pro-inflammatory statuses after SAH, the strength of communication interaction between monocyte subsets in CSF was significantly enhanced, suggesting that they depended on each other for biological functions. Consistent with our findings, a study reveals that CM highly express S100A8, S100A9, and S100A12, IM express HLA-DQA1 and HLA-DPA1 and NCM mainly express CD16, demonstrating a distinct transcriptome across monocyte subsets in Kawasaki disease (Geng et al., 2021). Similarly, in diabetic macular edema, intermediate monocytes express a high level of HLA-related genes, indicating a primary biological function of antigen processing and presentation (Ma et al., 2021). A study on gout demonstrates that CM and IM show similar differentially expressed genes (DEGs) while NCM primarily express the heat shock protein (HSP) family, which supports that CM and IM contribute to the immune response during gout flares (Yu et al., 2024). Another study also reveals that HLA-DR^low^S100A^high^ monocytes are related to late sepsis and show significant immunosuppressive function in immune response (Yao et al., 2023). These scRNA-seq studies demonstrate that monocyte subsets perform various roles in inflammation response with their characteristics.

Monocytes originate from bone marrow, then are released into the circulation system, and eventually undergo a series of differentiation into distinct subsets (Wolf et al., 2019). Monocytes are traditionally divided into three subsets based on the expression of marker genes (CD14 and CD16): CM, IM, and NCM (Ziegler-Heitbrock et al., 2010). Similarly, we defined monocytes as three subsets in SAH at a single cell level: CM, IM, and NCM. Furthermore, we found that monocyte subset differentiation was a gradual development process, namely, a lineage from CM by IM to NCM. Consistent with our findings, monocytes from patients with gout are also identified as CM, IM, and NCM (Yu et al., 2024). Monocytes from infants form three subsets with distinct pro-inflammatory gene signatures and biological functions. (Geng et al., 2021). Another study defines monocytes as CM, IM, and NCM in Prader-Willi syndrome (Xu et al., 2023b). These results suggest that scRNA-seq has advantages in studying cell clustering and differentiation development. Before the advent of scRNA-seq, it may be difficult to accurately describe the boundaries between these monocyte subsets using traditional experimental techniques.

There are some limitations in this study. First, these findings may need more representativeness due to the small sample size. In addition, considering thousands of monocytes in the circulatory system and CSF, the captured monocytes in our study may not be sufficient to reflect their roles in neuroinflammation. Finally, our results only represented the immune microenvironment at one point but not a dynamic inflammation process. Therefore, a study with a larger sample size is needed to confirm these findings.

In conclusion, we established a single-cell transcriptomic landscape of immune cells from CSF and PB in SAH and systematically analyzed monocyte heterogeneity in transcriptomic and phenotype. These findings highlighted that IM may play a key role in neuroinflammation after SAH by mediating the TLR and NLR pathways, thereby offering us new insight into the molecular mechanism of neuroinflammation and therapeutic targets for SAH.

## Data Availability

The data reported in this paper have been deposited in the Genome Sequence Archive for Human in National Genomics Data Center, China National Center for Bioinformation / Beijing Institute of Genomics, Chinese Academy of Sciences (GSA: HRA006959).
