# Supplementary material for: Single-cell transcriptomic landscape reveals the role of intermediate monocytes in aneurysmal subarachnoid hemorrhage
Source: Front Cell Dev Biol. 2024 Sep 10;12:1401573. doi: 10.3389/fcell.2024.1401573 (PMC11420033; doi:10.3389/fcell.2024.1401573)
Supplement: Supplementary file 1 [file Table1.docx]

| Clinical variable | **Case 1** | **Case 2** |
| --- | --- | --- |
| Age | 58 | 63 |
| Gender | male | male |
| Hunt & Hess score | 3 | 3 |
| Fisher score | 3 | 3 |
| Hypertension | Yes | Yes |
| Diabetes mellitus | No | No |

**Table S1**   **Baseline demographic and clinical data**

**Table S2 Baseline demographic and clinical data**

| Clinical variable | Healthy Controls (HC) |
| --- | --- |
| Age [years (mean ± SD)] | 72.52 ± 6.96 |
| Male (%) | 43 |
| Female (%) | 57 |

Note that the data are based on a cohort of healthy individuals.
