# Supplementary material for: Single-cell transcriptomic landscape reveals the role of intermediate monocytes in aneurysmal subarachnoid hemorrhage
Source: Front Cell Dev Biol. 2024 Sep 10;12:1401573. doi: 10.3389/fcell.2024.1401573 (PMC11420033; doi:10.3389/fcell.2024.1401573)
Supplement: Supplementary file 2 [file DataSheet2.docx]

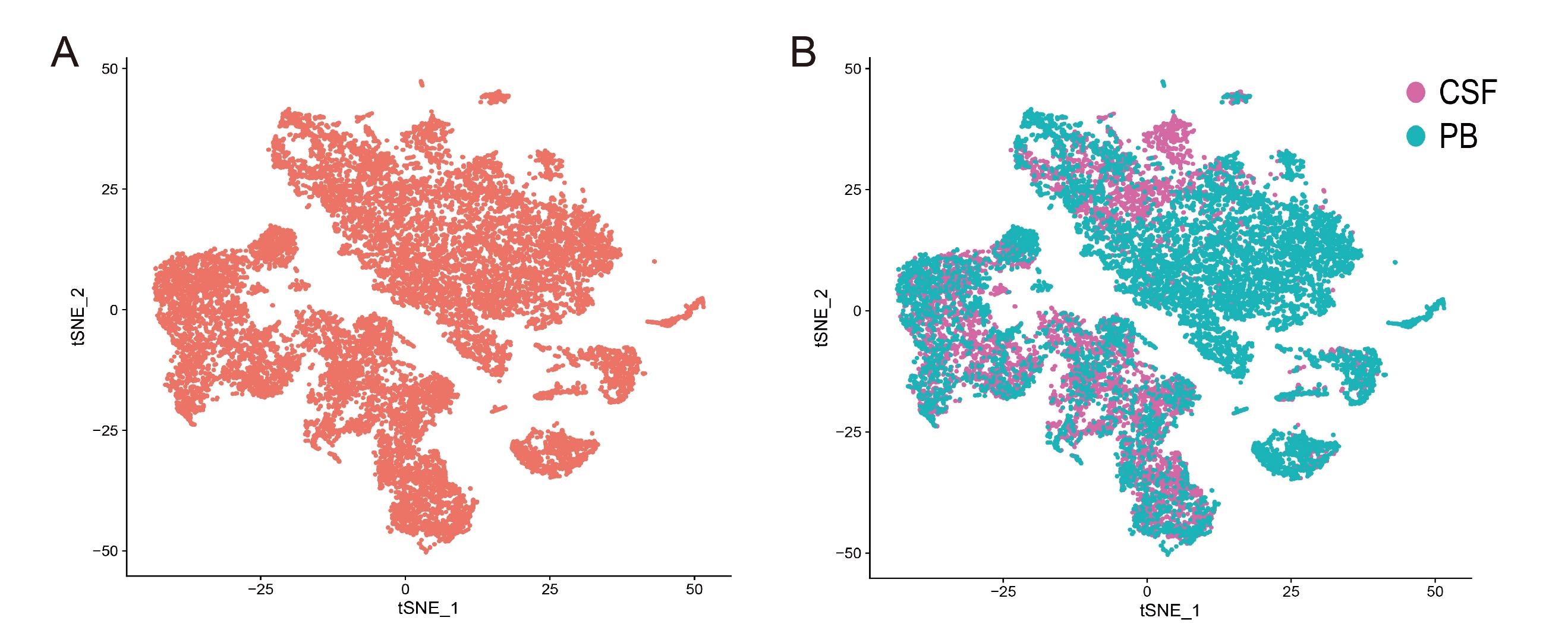


**Figure S1.** (A) T-SNE visualization of all cells from CSF and PB. (B) T-SNE visualization of cells in CSF and PB respectively.


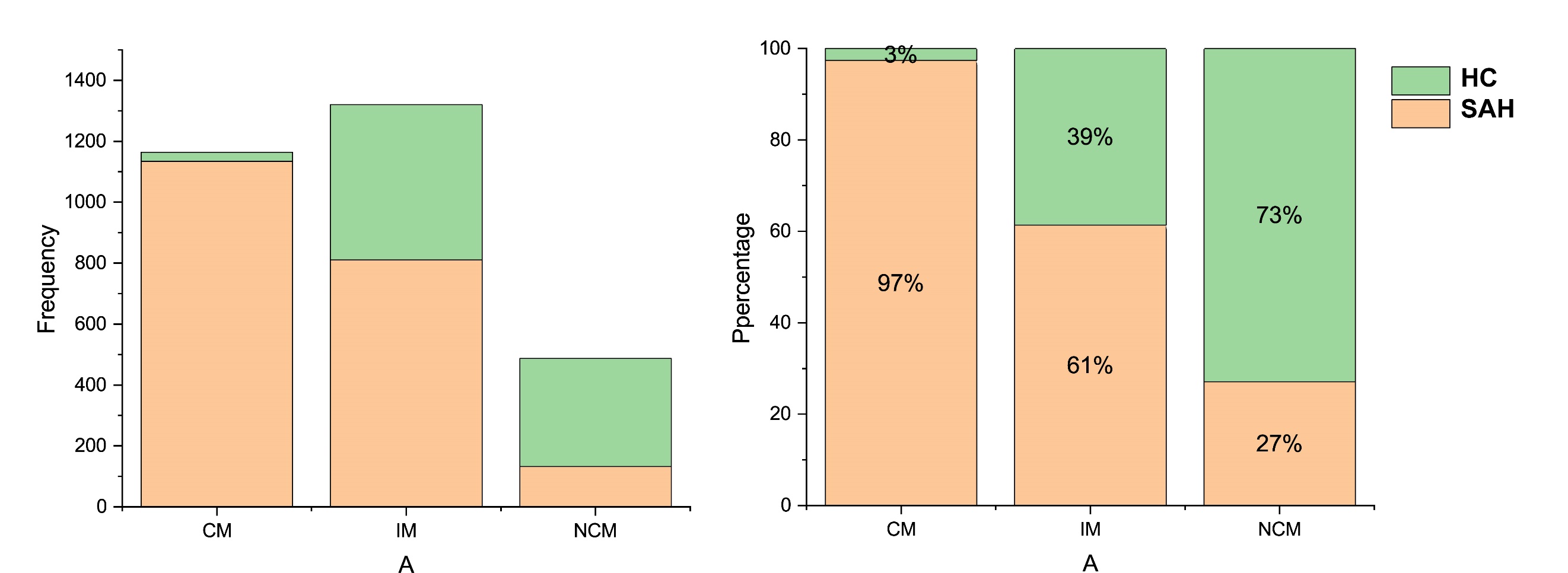


**Figure S2.** The total number and percentage of monocyte subsets from HC and SAH.
